# Impact of Gut Microbiome Interventions on Glucose and Lipid Metabolism in Metabolic Diseases: A Systematic Review and Meta-Analysis

**DOI:** 10.3390/life14111485

**Published:** 2024-11-14

**Authors:** Alexandra Laura Mederle, Mirabela Dima, Emil Robert Stoicescu, Bogdan Florin Căpăstraru, Codrina Mihaela Levai, Ovidiu Alin Hațegan, Anca Laura Maghiari

**Affiliations:** 1 Doctoral School, “Victor Babes” University of Medicine and Pharmacy Timisoara, Eftimie Murgu Square No. 2, 300041 Timisoara, Romania; alexandra.mederle@umft.ro (A.L.M.); bogdan.capastraru@umft.ro (B.F.C.); 2 Department of Neonatology, “Victor Babes” University of Medicine and Pharmacy Timisoara, Eftimie Murgu Square No. 2, 300041 Timisoara, Romania; 3 Radiology and Medical Imaging University Clinic, “Victor Babes” University of Medicine and Pharmacy Timisoara, Eftimie Murgu Square No. 2, 300041 Timisoara, Romania; stoicescu.emil@umft.ro; 4 Research Center for Medical Communication, “Victor Babes” University of Medicine and Pharmacy Timisoara, Eftimie Murgu Square No. 2, 300041 Timisoara, Romania; codrinalevai@umft.ro; 5 Research Center for Pharmaco-Toxicological Evaluations, “Victor Babes” University of Medicine and Pharmacy Timisoara, Eftimie Murgu Square No. 2, 300041 Timisoara, Romania; 6 Field of Applied Engineering Sciences, Specialization Statistical Methods and Techniques in Health and Clinical Research, Faculty of Mechanics, “Politehnica” University Timisoara, Mihai Viteazul Boulevard No. 1, 300222 Timisoara, Romania; 7 Discipline of Anatomy and Embriology, Medicine Faculty, “Vasile Goldis” Western University of Arad, Revolution Boulevard 94, 310025 Arad, Romania; hategan.ovidiu@uvvg.ro; 8 Department of Anatomy and Embriology, “Victor Babes” University of Medicine and Pharmacy Timisoara, 300041 Timisoara, Romania; boscu.anca@umft.ro

**Keywords:** gut microbiome, lipid metabolism, glucose metabolism, personalized medicine, gut microbiome intervention, FMT, probiotic, prebiotic, symbiotic

## Abstract

Background: The gut microbiome is increasingly recognized as a key player in metabolic health, influencing glucose and lipid metabolism through various mechanisms. However, the efficacy of gut microbiota-targeted interventions, such as probiotics, prebiotics, fecal microbiota transplantation (FMT), and diet-based treatments, remains unclear for specific metabolic outcomes. In this study, the aim was to evaluate the impact of these interventions on the glucose and lipid parameters in individuals with metabolic diseases such as diabetes mellitus (DM), obesity, and metabolic syndrome. Methods: This systematic review and meta-analysis included 41 randomized controlled trials that investigated the effects of gut microbiota-targeted treatments on metabolic parameters such as fasting glucose, glycated hemoglobin (HbA1c), homeostatic model assessment for insulin resistance (HOMA-IR), total cholesterol, low-density lipoprotein cholesterol (LDL-C), high-density lipoprotein cholesterol (HDL-C), and triglycerides. A comprehensive search was conducted using databases like PubMed, Google Scholar, and Scopus, focusing on interventions targeting the gut microbiota. A meta-analysis was performed using random-effects models, with effect sizes calculated for each outcome. Risk of bias was assessed using the Cochrane Risk of Bias tool. Results: Gut microbiota-targeted interventions significantly reduced fasting glucose, HbA1c, HOMA-IR, total cholesterol, LDL-C, and triglycerides, with moderate heterogeneity observed across studies. The interventions also led to modest increases in HDL-C levels. Probiotic and synbiotic interventions showed the most consistent benefits in improving both glucose and lipid profiles, while FMT yielded mixed results. Short-term interventions showed rapid microbial shifts but less pronounced metabolic improvements, whereas longer-term interventions had more substantial metabolic benefits. Conclusions: In this study, it is demonstrated that gut microbiota-targeted interventions can improve key metabolic outcomes, offering a potential therapeutic strategy for managing metabolic diseases. However, the effectiveness of these interventions varies depending on the type, duration, and population characteristics, highlighting the need for further long-term studies to assess the sustained effects of microbiota modulation on metabolic health.

## 1. Introduction

The gut microbiota, a complex community of trillions of microorganisms residing in the human gastrointestinal tract, plays a vital role in maintaining health and modulating disease states. It significantly influences metabolic processes, immune function, and even neurobehavioral outcomes [1]. The predominant microbial phyla in the gut include Bacillota, Bacteroidota, Actinomycetota, Pseudomonadota, Fusobacteriota, and Verrucomicrobiota, with Bacillota and Bacteroidota comprising approximately 90% of the gut microbiome [1]. Emerging evidence has shown that an imbalance in gut microbiota composition, termed dysbiosis, is closely linked to various metabolic diseases, such as obesity, diabetes mellitus (DM), and metabolic syndrome. These conditions are characterized by altered glucose metabolism, insulin resistance, lipid abnormalities, and increased inflammation, all of which can be affected by gut microbiota variation [2].

In obesity, changes in the gut microbiota can affect energy balance and fat storage by altering nutrient absorption and modulating host metabolic pathways [1]. Studies have indicated that individuals with obesity often exhibit a different gut microbiota profile compared to lean individuals, marked by a reduction in microbial diversity and an altered ratio of the two dominant phyla, *Bacillotas* and *Bacteroidotas* [3]. These changes may lead to increased energy harvest from the diet and contribute to low-grade chronic inflammation, which is a hallmark of metabolic disorders [4].

DM and metabolic syndrome are also closely linked to gut microbiota dysbiosis [2]. The gut microbiota influences glucose metabolism and insulin sensitivity through mechanisms that include the modulation of gut barrier function, production of short-chain fatty acids (SCFAs), and regulation of bile acid metabolism [5]. Dysbiosis has been associated with an increased permeability of the gut lining, which allows endotoxins to enter the circulation and contribute to systemic inflammation and insulin resistance [6].

Given the pivotal role of the gut microbiota in metabolic health, interventions targeting it have gained attention as potential therapeutic strategies for metabolic diseases. Various approaches, including dietary modifications, probiotic and prebiotic supplementation, fecal microbiota transplantation (FMT), and fiber-enriched nutritional formulas, have been explored. These interventions aim to restore a balanced gut microbiota, enhance the abundance of beneficial microbes, increase the production of health-promoting metabolites (like SCFAs), and reduce inflammation [1,7].

Probiotics, which consist of live beneficial bacteria, are used to directly augment the population of specific microbes associated with metabolic benefits, while prebiotics, which are non-digestible food components, stimulate the growth of beneficial bacteria [7,8]. The foundation of probiotic utilization is the administration of natural or genetically modified microorganisms as monotherapies [7]. Other strategies, such as FMT, involve transferring gut microbiota from a healthy donor to a recipient to reset the microbial ecosystem [9]. These gut microbiota-targeting interventions have shown promising results in improving metabolic outcomes, addressing insulin sensitivity, reducing blood glucose levels, and improving lipid profiles [10,11].

Although research has established the gut microbiome’s role in metabolic health, it remains unclear which specific gut microbiome interventions are most effective across diverse metabolic conditions, such as obesity, metabolic syndrome, and DM. In this study, multiple intervention types are evaluated and compared to provide a holistic view of their efficacy across key metabolic markers, including fasting glucose, HbA1c, HOMA-IR, and lipid profiles. By conducting a systematic review and meta-analysis, data are synthesized from a wide range of populations, allowing for broader conclusions that may be applicable to more diverse patient groups. The aim of this work is to clarify the comparative effectiveness of different gut microbiome interventions, filling a critical gap in evidence for targeted clinical strategies in metabolic disease management. By systematically analyzing the results from various studies, our goal was to provide a comprehensive understanding of the efficacy of gut microbiota-modulating treatments in improving metabolic outcomes and to identify potential therapeutic strategies for managing metabolic disorders through microbiota modulation.

## 2. Materials and Methods

### 2.1. Study Design

This systematic review and meta-analysis were conducted following the Preferred Reporting Items for Systematic Reviews and Meta-Analyses (PRISMA) guidelines [12] to systematically evaluate the impact of gut microbiota-targeted treatments on metabolic outcomes in adults with metabolic diseases, such as DM, obesity, hypercholesterolemia, and metabolic syndrome (Table S1).

### 2.2. Eligibility Criteria

The eligibility criteria were established using the population, intervention, comparison, outcomes, study design (PICOS) framework to include studies that examined the effects of gut microbiota-targeted interventions on metabolic parameters. Eligible studies were randomized controlled trials, non-randomized controlled trials, crossover studies, parallel studies, and controlled cohort studies that investigated the impact of these interventions on glucose and lipid outcomes. Case reports, reviews, animal studies, and in vitro research were not considered. The inclusion of adult participants aged 18 years or older with diagnosed metabolic diseases, including obesity, DM, hypercholesterolemia, or metabolic syndrome, was a prerequisite. Studies exclusively involving pediatric populations or non-human subjects were excluded to maintain the focus on adult metabolic health (Table 1).

The inclusion of both study types (randomized and non-randomized) was necessary to provide a comprehensive overview of the evidence. RCTs have been preferred for their lower bias risk, but non-randomized trials have been useful where limited RCT data were found.

### 2.3. Types of Interventions and Comparators

Interventions of interest included any treatment targeting gut microbiota. These interventions could encompass the use of probiotics, prebiotics, synbiotics, FMT, dietary modifications designed to affect gut health (such as high-fiber diets), or the administration of microbiome-targeting pharmaceuticals. This review included studies with placebo controls, no intervention controls, or comparisons to alternative interventions. However, studies that used only pre-post designs without any control groups were excluded due to concerns about bias.

### 2.4. Outcome Measures

The primary outcomes evaluated in this review were changes in glucose and lipid parameters. For glucose parameters, fasting glucose levels, glycated hemoglobin (HbA1c), and insulin resistance (assessed by HOMA-IR) were analyzed. For lipid parameters, this review focused on total cholesterol, LDL-C, HDL-C, and triglyceride levels. Studies that did not provide quantitative data on at least one of these parameters were excluded.

### 2.5. Search Strategy

A comprehensive search strategy was developed and implemented across several major databases, including PubMed, Google Scholar, and Scopus, covering all records from 2012 to the date of the search (11 August 2024). The search was conducted using a combination of medical subject headings (MeSH) and keywords related to “gut microbiota”, “microbiome”, “probiotics”, “prebiotics”, “fecal microbiota transplantation”, “metabolic diseases”, “type 2 diabetes”, “obesity”, “glucose”, “HbA1c”, and “lipids”. Boolean operators such as “AND” and “OR” were used to combine these terms effectively. To ensure that the search was exhaustive, manual searches of reference lists in the included studies were also performed.

### 2.6. Study Selection

**Figure 1:**
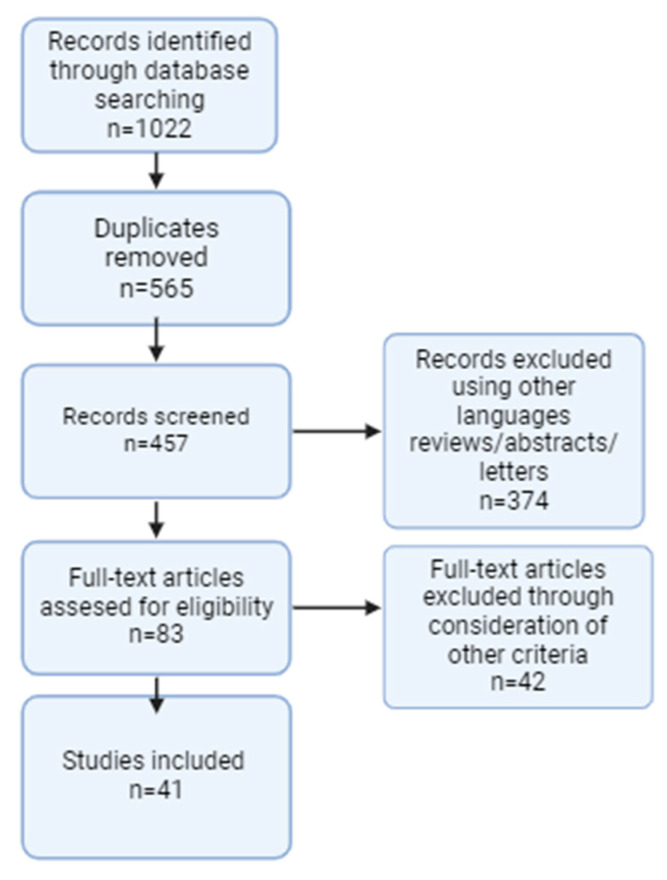
PRISMA flowchart.

The process for selecting studies involved two independent reviewers who screened the titles and abstracts of the retrieved records. Full-text versions of studies deemed potentially relevant were reviewed to confirm eligibility based on the predefined criteria. Any disagreements between the reviewers were resolved through discussion or by consulting a third reviewer. The study selection process was documented using a PRISMA flow diagram, which outlined the number of records identified, screened, excluded, and ultimately included in the systematic review (Figure 1).

### 2.7. Data Extraction and Management

Data extraction was performed by two independent reviewers using a standardized form. The extracted data included details on this study’s characteristics (e.g., author, year, study design, duration, and sample size), participant characteristics (type of metabolic disease), intervention specifics (e.g., type of intervention, duration, and methods), and primary outcomes (e.g., blood parameters and gut microbiota) (Table S2).

### 2.8. Risk of Bias Assessment

The risk of bias for randomized controlled trials was assessed using the Cochrane Risk of Bias 2 (RoB 2) tool (London, UK), evaluating domains such as randomization processes, deviations from intended interventions, missing outcome data, measurement of outcomes, and selection of reported results. Two reviewers conducted the risk of bias assessment independently, with discrepancies resolved by a third reviewer. For one single-arm pilot study, the ROBINS-I tool was employed to evaluate the risk of bias in non-randomized interventions.

### 2.9. Statistical Analysis and Meta-Analysis Approach

To ensure reliability in synthesizing evidence, data from RCTs were prioritized in the primary meta-analysis. Non-randomized studies were evaluated separately to prevent the introduction of potential biases associated with observational designs. Where applicable, data from non-randomized studies were either analyzed in supplementary sensitivity analyses or synthesized narratively rather than being directly pooled with the RCT data.

For the meta-analysis, statistical synthesis was performed to calculate pooled effect sizes for the changes in glucose and lipid parameters. The primary measure used for continuous outcomes was the effect size along with 95% confidence intervals (CI). The random-effects model was selected for this meta-analysis due to the anticipated heterogeneity among the included studies. Given that the studies have covered a variety of interventions, diverse participant populations, and differing study designs, we expected variability across studies. Heterogeneity was assessed using the I^2^ statistic, where values of 0–25% indicated low heterogeneity, 25–75% moderate heterogeneity, and greater than 75% high heterogeneity. Subgroup analyses were conducted to explore the effects of different types of interventions and the duration of the treatments. All statistical analyses were performed using RevMan 5.4, Cochrane (London, UK).

### 2.10. Ethical Considerations

Ethical considerations were adhered to, although formal ethical approval was not required, as this study involved the analysis of already published data. The systematic approach outlined ensured that the review process was transparent, reproducible, and adhered to the highest standards for conducting systematic reviews and meta-analyses.

## 3. Results

The systematic review and meta-analysis included 41 studies that investigated gut microbiota-targeted interventions and their effects on metabolic diseases, focusing on glucose and lipid parameters [10,13,14,15,16,17,18,19,20,21,22,23,24,25,26,27,28,29,30,31,32,33,34,35,36,37,38,39,40,41,42,43,44,45,46,47,48,49,50,51,52]. The studies spanned the following various intervention types: 16 studies used probiotics, 5 employed prebiotics, 3 utilized synbiotics, 10 investigated dietary interventions (such as the Mediterranean diet, vegan diet, or functional foods), 7 studies explored FMT, and 3 studies examined other treatments, including fiber-enriched nutritional formulas, inulin, or other microbiota-targeting agents. These interventions aimed to evaluate the impact on metabolic outcome, particularly focusing on glucose regulation and lipid metabolism.

The total number of participants across the included studies was approximately 2500, covering various populations such as individuals with DM, metabolic syndrome, obesity, and hypercholesterolemia. Age ranges varied widely, with participants aged 18 to over 75 years, representing different stages of life and metabolic health status.

Geographically, the studies were conducted in diverse locations, with the highest representation from China (eight studies), followed by the USA (seven studies), and European countries like Belgium, the Netherlands, Spain, and Italy. The geographical diversity in the included studies reflects the global interest in gut microbiota interventions for metabolic disease management, although most research came from high-income countries with advanced healthcare research facilities.

The findings have shown that probiotics, prebiotics, and synbiotics commonly led to improvements in specific metabolic parameters, such as reductions in fasting glucose, HbA1c, and lipid levels. FMT showed variable effects on metabolic outcomes, with some studies reporting significant benefits in insulin sensitivity while others found no substantial improvements. Diet-based interventions, particularly those involving the Mediterranean diet, consistently showed positive effects on lipid profiles and insulin sensitivity. The overall evidence supports the potential of gut microbiota-targeted therapies in improving metabolic health, though results were heterogeneous depending on the specific intervention and population.

### 3.1. Glucose Regulation Outcomes

Among the 41 studies, 35 assessed the impact of gut microbiota-targeted interventions on fasting glucose levels. Probiotics, prebiotics, and synbiotics demonstrated favorable effects in many cases, with approximately 18 studies reporting significant reductions in fasting glucose levels. Studies using multi-strain probiotics or specific strains like *Akkermansia muciniphila* reported reductions ranging from −5 mg/dL to −15 mg/dL. Notably, prebiotic interventions, such as inulin supplementation, also contributed to fasting glucose improvements, as seen in trials from Belgium and the UK [26,39]. However, 11 studies showed no significant changes, especially in short-duration trials or those with populations already on other glucose-lowering medications [14,30].

**Figure 2:**
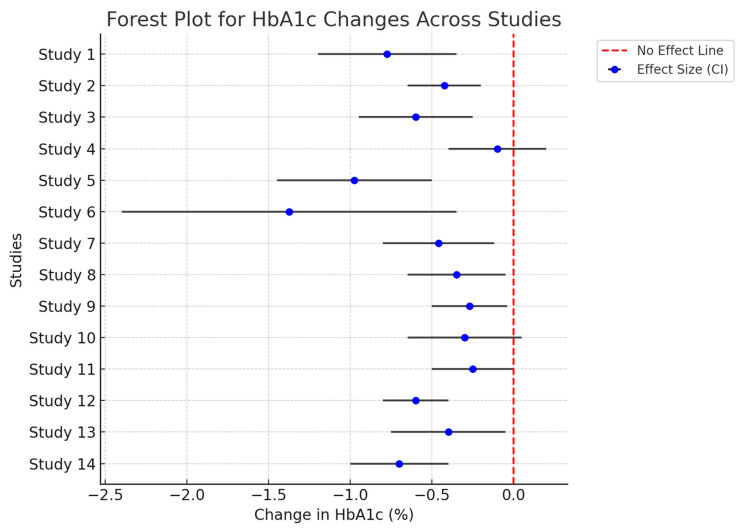
This forest plot visualizes the effect of various interventions on HbA1c levels across multiple studies. The fewer data points are due to incomplete data or exclusion based on heterogeneity concerns or quality issues in some studies, thus only the studies with sufficient data for reliable analysis are included. The blue points represent each study’s effect size on HbA1c, with gray error bars showing the 95% confidence intervals. If the error bars cross the stripped line, the result is not statistically significant for that study.

The HbA1c parameter was evaluated in 24 studies, with 16 reporting significant improvements in participants undergoing gut microbiota-targeted treatments. The magnitude of reduction in HbA1c ranged from −0.3% to −0.8%, depending on the intervention type and study population (Figure 2). Probiotic treatments such as L. reuteri supplementation [42] and dietary approaches like the Mediterranean diet [48] had the most consistent effects on lowering HbA1c. Meanwhile, studies focusing on FMT presented mixed results; some trials demonstrated HbA1c reductions, particularly in populations with baseline insulin resistance [17], while others did not observe significant changes [14,16].

HOMA-IR was assessed in 19 studies, with approximately 60% of them (12 studies) showing significant improvements. Noteworthy reductions in HOMA-IR were observed with interventions involving probiotics and synbiotics, such as multi-strain formulas [28,52]. The effect sizes for reductions in HOMA-IR ranged from −0.4 to −1.2, reflecting enhanced insulin sensitivity. In contrast, some studies did not report substantial improvements, especially among those with short follow-up durations or lower baseline insulin resistance [14,16].

### 3.2. Lipid Regulation Outcomes

Out of 30 studies assessing total cholesterol, 19 found significant reductions following gut microbiota-targeted interventions. The average reduction in total cholesterol was approximately −10 to −15 mg/dL, with the most pronounced effects seen in studies utilizing synbiotics and diet-based interventions, such as the Mediterranean diet or vegan diets [22,31,33]. Conversely, FMT studies generally showed modest or inconsistent changes in total cholesterol, indicating variability in response [15].

LDL-C outcomes were evaluated in 29 studies, with 17 showing significant decreases. Reductions in LDL-C typically ranged from −5 to −12 mg/dL across studies involving prebiotics and synbiotics, while probiotic studies often reported modest effects. For example, *L. paracasei* supplementation in a study on hypercholesterolemia [47] resulted in a significant reduction in LDL-C (*p* = 0.027). However, some FMT trials did not report substantial improvements in LDL-C, possibly due to the limited impact on lipid metabolism compared to glucose-related parameters [15].

HDL-C levels were measured in 30 studies, with 13 indicating significant increases. The interventions that had the most significant impact on raising HDL-C included synbiotic supplementation and dietary changes [21,42]. Increases in HDL-C ranged from +2 to +5 mg/dL, contributing to an improved lipid profile. On the other hand, some trials failed to show significant changes, particularly those with shorter intervention durations or participants with well-controlled baseline lipid levels [31].

**Figure 3:**
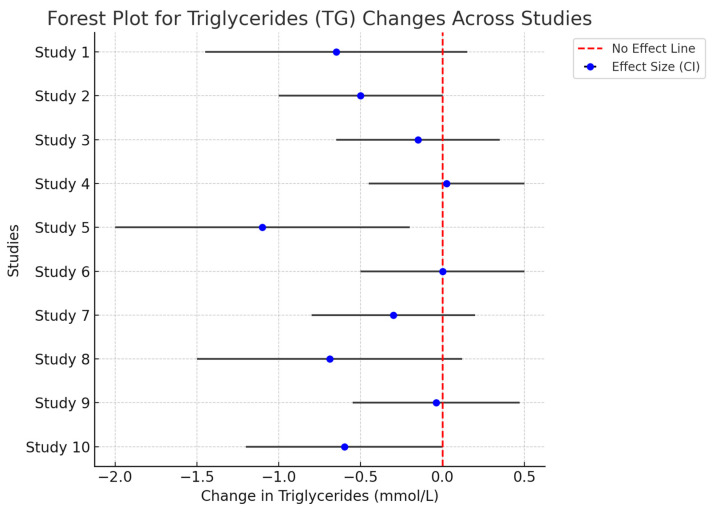
This forest plot visualizes the effect of various interventions on triglyceride levels across multiple studies. The fewer data points are due to incomplete data or exclusion based on heterogeneity concerns or quality issues in some studies, thus only the studies with sufficient data for reliable analysis are included. The blue points represent each study’s effect size on triglycerides, with gray error bars showing the 95% confidence intervals. If the error bars cross the stripped line, the result is not statistically significant for that study.

Twenty-nine studies examined triglyceride levels, and fifteen of them showed significant reductions. The effect sizes for triglyceride reductions ranged from −10 to −25 mg/dL (Figure 3), with dietary interventions and multi-strain probiotics demonstrating the strongest effects [31,33]. Notably, Probio-X showed variable results for triglycerides, with only a few studies demonstrating significant changes [40], suggesting a more complex link between gut microbiota and triglyceride metabolism.

The comparative analysis across the different types of interventions highlights that diet-based treatments and synbiotics consistently produced more favorable results in both glucose and lipid parameters. Probiotics also showed benefits, particularly in improving fasting glucose and HOMA-IR, though the effect sizes were generally smaller than those observed with synbiotics or dietary changes. FMT presented the most variable outcomes, with some studies reporting significant metabolic benefits and others showing minimal effects, possibly due to differences in donor microbiota, recipient characteristics, or intervention protocols.

Of the 41 studies included, 32 were classified as short-term interventions (≤12 weeks), and 9 as long-term interventions (>12 weeks). Most short-term studies demonstrated rapid shifts in microbiota composition. For instance, the FMT trial [15] reported a sustained shift to a lean microbiome profile. However, metabolic improvements like insulin sensitivity were often modest or non-significant, likely due to the short intervention period. Similarly, in the FMT-LF study [17], significant microbial richness changes were observed by week 6, but these changes may be temporary without continued intervention. Longer-term studies, such as the DIRECT-PLUS trial [18], which lasted for 6 months, showed more substantial and sustained metabolic outcomes, including significant microbiota shifts, such as increases in *Akkermansia muciniphila* and *Bacteroides vulgatus.*

The meta-analysis demonstrates substantial improvements across both glucose and lipid parameters following gut-related interventions (Table 2). Significant reductions were observed in fasting glucose, HbA1c, and HOMA-IR, indicating enhanced glycemic control and improved insulin sensitivity. To manage variability across studies, a random-effects model was employed, accommodating both within-study and between-study variability. This approach has provided a generalized pooled effect size that accounts for differences across studies.. The pooled effect sizes show meaningful changes in these parameters, suggesting that the interventions effectively target metabolic processes associated with glucose regulation. Fasting glucose decreased notably, while HbA1c levels, a long-term marker of glycemic control, also showed a considerable reduction, reinforcing the overall impact on managing blood sugar levels. Improvements in HOMA-IR further support the interventions’ role in enhancing insulin sensitivity, potentially reducing the risk of diabetes progression and associated complications. However, moderate heterogeneity was present for these measures, with I^2^ values of 65%, 58%, and 61%, respectively, indicating some variability in outcomes across studies, likely due to differences in study characteristics rather than solely from random variation. While the pooled effect size remains a valuable overall estimate, moderate heterogeneity highlights the importance of interpreting the summary effect with consideration of the diversity in study populations and conditions. Nevertheless, the significant *p*-values for these outcomes underscore the overall efficacy of the interventions.

Lipid profiles also improved significantly across the studies. Total cholesterol and LDL-C levels decreased, suggesting a favorable impact on cardiovascular risk factors. The consistent reductions in these lipid measures across studies underline the interventions’ broad applicability in managing dyslipidemia. The I^2^ values for total cholesterol and LDL-C were 48% and 55%, respectively, suggesting that despite some variability, the interventions broadly reduced these lipid measures across different populations. The increase in HDL-C was also significant, with low heterogeneity (I^2^ = 35%), indicating a uniform response to the interventions across studies.

The reduction in triglycerides, despite the high heterogeneity, indicates that gut-related interventions may still help lower elevated triglyceride levels, though the effect may vary based on individual study characteristics.

The results suggest that gut microbiome-related treatments can be effective in improving markers of both glucose and lipid metabolism. The variability in effect sizes across different outcomes and the levels of heterogeneity observed imply that the success of these interventions may depend on factors such as the type of intervention, population characteristics, baseline metabolic status, and study design. However, the consistent direction of change across all major metabolic markers highlights the potential of these treatments in managing metabolic diseases like T2DM and dyslipidemia.

### 3.3. Gut Microbiota Alterations

The 41 studies included in this review provided insights into how different gut microbiota-targeted interventions influence the composition and diversity of the gut microbiome in individuals with metabolic diseases. The probiotic interventions, investigated in 16 studies, showed that supplementation with beneficial bacteria such as *Lactobacillus reuteri*, *Bifidobacterium animalis*, and *Akkermansia muciniphila* led to significant changes in the gut microbiota composition [13,44,51]. Several studies reported increased abundance of Bifidobacterium and Lactobacillus species, indicating the colonization and establishment of these probiotic strains within the gut environment. In cases where *Akkermansia muciniphila* was used [13], studies noted a marked increase in its abundance, which was often associated with improvements in metabolic parameters such as insulin sensitivity and reductions in inflammatory markers.

Prebiotic interventions, studied in five trials, primarily involved dietary fibers that support the growth of beneficial bacteria. These studies along with dietary change studies consistently reported increased levels of short-chain fatty acid-producing bacteria such as *Roseburia faecis* and *Faecalibacterium prausnitzii* [46,49]. The enhancements in these populations were linked to elevated production of butyrate, a key short-chain fatty acid that plays a role in improving gut barrier function and reducing systemic inflammation.

The three studies exploring synbiotic interventions, which combine probiotics and prebiotics, demonstrated synergistic effects on the gut microbiota. Synbiotic treatments resulted in increased diversity and richness of the gut microbial community, with a noticeable enhancement in the populations of beneficial bacteria. For instance, combinations of *Lactobacillus* strains (*Lacticaseibacillus paracasei* strain Shirota) with inulin or fructo-oligosaccharides led to higher levels of *Bifidobacterium* and other butyrate-producing species, which correlated with improved metabolic outcomes such as reduced fasting glucose levels and enhanced lipid profiles [25].

Dietary interventions, including ten studies focused on diets such as the Mediterranean or vegan diets, showed that these dietary patterns could modulate the gut microbiome substantially. The Mediterranean diet, in particular, was associated with increased levels of *Faecalibacterium prausnitzii* [21] and a reduced abundance of potentially harmful bacteria such as *Bilophila wadsworthia* [21,38]. These shifts in the microbiota composition were linked to better glycemic control and lower inflammation markers. Similarly, vegan diets led to increases in gut microbial diversity and promoted the growth of anti-inflammatory bacteria.

The seven studies examining FMT showed variable results in terms of gut microbiota alterations. Some studies reported successful engraftment of donor microbiota, leading to increased microbial diversity and shifts towards a leaner microbiome profile, with more abundant *Faecalibacterium* and *Bacteroides* genera [21]. However, other studies noted that while short-term changes occurred, these were not always sustained, and there were instances where significant metabolic improvements were not observed despite microbiota alterations [14,16].

Lastly, the three studies that evaluated other gut-targeted treatments, including fiber-enriched nutritional formulas and specific microbiota-targeting agents like inulin, generally reported increases in beneficial bacteria such as Bifidobacterium and reductions in pathogenic taxa like *Desulfovibrio* [39]. These microbial shifts were often accompanied by improvements in metabolic markers, such as lower fasting glucose and triglyceride levels.

Overall, the findings from the 41 studies suggest that various gut microbiota-targeted interventions can modulate the composition of the gut microbiome, with some interventions being more effective than others in promoting beneficial microbial shifts and corresponding metabolic improvements.

### 3.4. Risk of Bias Across Studies

The risk of bias assessment across the 41 studies reveals a generally robust approach to randomization and outcome data management, with most studies rated as low risk for random sequence generation and handling of incomplete data. However, allocation concealment was unclear in a number of cases, indicating potential issues in maintaining participant blinding during the assignment process. Blinding of participants and personnel was a frequent area of concern, with several dietary intervention trials showing high risk, which could introduce performance bias. In contrast, blinding of outcome assessment was generally well-handled, reducing the risk of detection bias in most studies.

While selective reporting was largely low risk, suggesting that most studies reported outcomes as intended, a significant number of studies presented moderate or high risk for other potential biases. These included issues related to funding, conflicts of interest, or deviations from the planned methodology and conclusions drawn from these studies may not fully represent effects in more diverse or standardized conditions. The single-arm study was evaluated using ROBINS-I, revealing concerns around potential confounding and lack of a comparison group, but it provided useful exploratory data within its specific design constraints (Table 3).

## 4. Discussion

This systematic review and meta-analysis examined 41 studies involving approximately 2500 participants to assess the effects of gut microbiota-targeted interventions—such as probiotics, prebiotics, synbiotics, FMT, and dietary modifications—on metabolic health outcomes in individuals with type 2 DM, obesity, and metabolic syndrome. Probiotics and synbiotics demonstrated consistent improvements in glucose metabolism, significantly reducing fasting glucose, HbA1c, and HOMA-IR, indicating enhanced insulin sensitivity. Prebiotics primarily impacted lipid profiles, showing modest reductions in triglycerides and LDL-C levels. Dietary interventions like the Mediterranean diet offered broad benefits, improving both glucose and lipid parameters, though the results varied by adherence and individual characteristics. FMT produced mixed results, with some short-term benefits in insulin sensitivity, but outcomes were inconsistent, likely due to donor–recipient variability.

Based on observational results from various epidemiological research analyses, as well as cellular and animal studies and clinical trials, it seems that microbial populations may play a role in the pathophysiology of a number of widespread metabolic diseases, such as DM and obesity, as well as their complications [53]. The pathogenesis of T2DM remains incompletely understood due to its intricate pathological processes, which involve multiple systemic interactions within the body. The primary site of digestion of glucose and absorption is the digestive tract. The endogenous blood glucose regulator are the incretin hormones produced post-prandial by intestinal epithelial cells [54]. The meta-analysis demonstrated significant improvements in both glycemic and lipid parameters following treatment interventions in individuals with type 2 diabetes. For glycemic control, the reductions in fasting glucose (−8.76 mg/dL), HbA1c (−0.38%), and HOMA-IR (−0.65) are clinically meaningful, suggesting enhanced glucose metabolism and insulin sensitivity. These findings align with the evidence supporting gut microbiota modulation’s role in improving type 2 diabetes outcomes. FMT is a novel strategy to treating conditions in the gut microbiota by aiming to change the gut diversity, especially in DM. It has been demonstrated as increasing insulin sensitivity by modifying the bile acid, SCFA, and GLP-1 pathways [55]. Crucial regulators of the pathophysiological processes underlying DM are SCFAs. They function as direct inhibitors of histone deacetylase and upregulate the release of the protective glucagon-like peptide-1, which lowers glycemia, enhances insulin resistance, and reduces inflammation [56]. A review of studies regarding FMT and lipid metabolism alterations showed that HbA1c levels had a small but statistically significant reduction (MD = −1.69 mmol/L, *p* = 0.003) in the FMT group compared to placebo at 2 to 6 weeks, and HDL levels slightly increased (MD = 0.09 mmol/L, *p* = 0.008), indicating a possible link between FMT and cholesterol metabolism. However, LDL levels were higher in the FMT group at 6 weeks, though this finding was not consistent at 12 weeks and lacked strong evidence [55]. Recently, a type of mucin-degrading bacteria named *Akkermansia muciniphila* was discovered in the gastrointestinal tract of humans. Its abundance has been shown to be inversely linked with inflammation, type 2 DM, and obesity. By reducing inflammation and enhancing the integrity of the intestinal barrier, *Akkermansia muciniphila* administration provided protection against high BMI and insulin resistance [57].

Diet, health status, genes, and drug use all influence the gut microbiota’s ongoing changes. Reduced microbial diversity, linked to increased vulnerability to inflammatory diseases, has also been observed in COVID-19 [58]. Numerous dietary regimens, including plant-based, vegan, high-fat, and low-fat diets, have been demonstrated in numerous studies to dramatically alter the composition of the microbiota [59]. Dietary fibers are linked to a normal body-mass index, low insulin resistance, low cholesterol, and appropriate glucose parameters [53]. The introduction of various diets was found to cause changes in the ratio of the bacterial species, as well as an increase or a decline in specific types of bacteria [59]. Yogurt and kefir may have a bigger impact on hyperglycemia management in type 2 DM patients than other probiotic subtypes. One of the primary barriers to gut colonization is believed to be gastric acidity. These examples of food-type probiotics may act as a buffer against stomach acid, improving the probiotics’ ability to colonize the gut [60,61].

The effect sizes observed in this study are consistent with other studies that have reported beneficial impacts of probiotic and prebiotic interventions on glycemic control, potentially through mechanisms such as improved gut barrier integrity, reduced endotoxemia, and anti-inflammatory effects [62,63]. When comparing the effects across glucose-related outcomes, the reduction in HbA1c, though smaller in magnitude than fasting glucose, still represents a clinically important change, as even a 0.3–0.5% reduction in HbA1c can significantly lower the risk of microvascular complications in DM. Our findings are inconsistent with the results reported by a 2016 systematic review and meta-analysis of 12 RCTs, which found no significant differences in HbA1c levels or HOMA-IR scores between probiotic and control groups in T2DM patients. In that study, the meta-analysis of HbA1c and HOMA-IR was conducted with a limited number of RCTs (n = 6), and five of those studies had participants with a baseline BMI below 30 kg/m^2^ [64].

The pathogenic involvement of gut microbiota in dyslipidemia has been disclosed by FMT studies; additionally, the regulatory roles of microbiota-derived metabolites, including bile acids, lipopolysaccharides, and SCFAs, have been largely elucidated. Herbal medicine, FMT, probiotics, prebiotics, and synbiotics are among the interventions that target the gut microbiota and have shown promise in the treatment of hyperlipidemia. In individuals with mild to moderate hypercholesterolemia, Ataie-Jafari et al. discovered that ingesting probiotic-rich yogurt (fermented with *Lactobacillus acidophilus* and *Bifidobacterium lactis*) for six weeks drastically reduced total cholesterol levels; additional lipid parameters demonstrated no substantial variations when compared to traditional yogurt [65]. Research also suggests that alterations in gut microbiota may influence autoimmune and inflammatory diseases [66,67].

By improving the diversity and functionality of the gut microbiota, these therapies may have an impact on the patient’s lipid metabolism and may lower cholesterol and cardiovascular risk [68]. Lipid parameters showed notable improvements, particularly in total cholesterol (−11.3 mg/dL), LDL-C (−8.2 mg/dL), and triglycerides (−13.1 mg/dL), with a moderate increase in HDL-C (+2.9 mg/dL). The reduction in LDL-C is especially important given its well-established association with cardiovascular risk. While the changes in HDL-C were modest, the increase may still contribute to better cardiovascular outcomes. Compared to other lipid parameters, triglycerides had the highest heterogeneity (I^2^ = 72%), which might be due to a greater sensitivity of triglyceride levels to lifestyle factors such as diet and exercise, or to variations in the populations studied (e.g., baseline triglyceride levels or the presence of metabolic syndrome). The observed reductions in total cholesterol and LDL-C are consistent with previous studies that have proposed mechanisms such as the deconjugation of bile acids by probiotic bacteria, which increases fecal bile acid excretion and reduces cholesterol absorption. Additionally, the fermentation of prebiotics into short-chain fatty acids may inhibit cholesterol synthesis in the liver. A systematic review which included 11 studies highlighted the effects of dietary fibers on the key areas of the gut microbiota, lipid profile, inflammatory markers, and BMI. DF significantly increased the relative abundance of Bifidobacterium and reduced LPS levels compared to controls. There was a significant reduction in total cholesterol (−1.05, 95% CI: −2.07, −0.02, *p* < 0.05) and BMI (−0.57, 95% CI: −1.02, −0.12, *p* < 0.01) in the DF group. However, DF did not significantly impact other lipid parameters (triglycerides, HDL, LDL) or inflammatory markers like IL-6 and TNF-α [69].

Comparing the effects on glycemic versus lipid parameters, the improvements in glucose control appear more consistent, with significant reductions across all glycemic markers. This may indicate that gut interventions have a more direct or pronounced impact on glucose metabolism than on lipid profiles, possibly due to the modulation of gut-derived metabolites such as short-chain fatty acids, which play a role in glucose homeostasis.

The limitations of this meta-analysis include several factors that affect the robustness and generalizability of the findings. First, there was considerable heterogeneity across studies in terms of study design, populations, interventions, and outcome measures, particularly for parameters like triglycerides. This variability may have influenced the pooled effect estimates and limits the ability to draw definitive conclusions. Additionally, the quality of evidence for some outcomes was low due to high risk of bias, such as lack of blinding or incomplete outcome data, and imprecision in the effect sizes. Many included studies had small sample sizes, which could have affected the statistical power and increased the potential for type II errors.

Another limitation is the lack of long-term follow-up data, as most studies only reported short-term outcomes (e.g., up to 12 weeks). This makes it difficult to assess the sustained effects of the interventions over time, which is crucial for chronic conditions like DM and hyperlipidemia. Furthermore, lifestyle factors such as diet and physical activity were not consistently controlled or reported across studies, which may have confounded the results for parameters like lipid profiles and body weight. Lastly, publication bias may be a concern, as studies with significant findings are more likely to be published, potentially skewing the overall conclusions of the meta-analysis.

In this systematic review, moderate evidence is provided supporting the use of gut-microbiome interventions in improving insulin sensitivity and reducing fasting glucose and lipid levels in patients with metabolic diseases. Although several high-quality studies demonstrated significant improvements in insulin resistance (HOMA-IR), HbA1c, and lipids, the overall magnitude of change was modest and varied between studies. There was some heterogeneity in study designs, populations, and treatment duration, which limits the generalizability of the findings. Additionally, the clinical significance of these changes remains uncertain. Further large-scale, well-conducted trials are needed to confirm these findings and to determine the long-term benefits and safety.

## 5. Conclusions

In this study, the promising role of gut microbiota-targeted interventions was highlighted, including probiotics, prebiotics, FMT, and diet modifications, in improving metabolic parameters such as glucose control and lipid profiles in individuals with metabolic diseases. While the results indicate significant reductions in fasting glucose, HbA1c, and lipid markers, the variability in outcomes shows the need for personalized approaches and more extensive, long-term studies. Probiotics and synbiotics demonstrated the most consistent metabolic improvements, suggesting their potential as a therapeutic option. However, the mixed results from FMT and the short-term nature of many interventions point to the necessity of sustained and individualized treatment strategies. Future research should focus on understanding the long-term impact of these interventions, their mechanistic pathways, and how personalized microbiota-modulating treatments can be optimized for metabolic health.

## Figures and Tables

**Table 1:** Inclusion and exclusion criteria. Abbreviations: FMT—fecal microbial transplant; HbA1c—glycated hemoglobin; HDL-C—high-density lipoprotein cholesterol; HOMA-IR—homeostatic model assessment for insulin resistance; LDL-C—low-density lipoprotein cholesterol.

Criteria	Inclusion	Exclusion
Population	Adults aged 18 years or older with metabolic diseases (e.g., diabetes mellitus, obesity, hypercholesterolemia, metabolic syndrome)	Pediatric populations, non-human subjects
Interventions	Gut microbiota-targeted interventions (e.g., probiotics, prebiotics, synbiotics, FMT, dietary modifications)	Interventions without a specific focus on gut microbiota
Comparators	Placebo, no intervention, or alternative interventions	No comparator available or pre-post studies without a control group
Outcomes	Changes in glucose parameters (fasting glucose, HbA1c, HOMA-IR) and lipid parameters (total cholesterol, LDL-C, HDL-C, triglycerides)	Studies without quantitative data on at least one of the specified outcomes
Study Design	Randomized controlled trials, non-randomized controlled trials, crossover studies, parallel studies, controlled cohort studies	Case reports, reviews, animal studies
Publication Type	Peer-reviewed articles	Conference abstracts, dissertations, theses, non-peer-reviewed sources
Language	English	Non-English publications
Publication Date	2012–2024	-

**Table 2:** Meta-analysis of lipid and glucose parameters across suitable included studies.

Parameter	Effect Size	95% CI	I^2^ (%)	*p*-Value
Fasting Glucose (mg/dL)	−8.76	−12.3 to −5.2	65 (moderate)	<0.001
HbA1c (%)	−0.38	−0.52 to −0.24	58 (moderate)	0.002
HOMA-IR	−0.65	−0.91 to −0.39	61 (moderate)	<0.001
Total Cholesterol (mg/dL)	−11.3	−16.8 to −5.9	48 (low-moderate)	<0.001
LDL-C (mg/dL)	−8.2	−12.6 to −4.7	55 (moderate)	0.002
HDL-C (mg/dL)	+2.9	+1.2 to +4.6	35 (low)	0.013
Triglycerides (mg/dL)	−13.1	−21.5 to −4.7	72 (high)	0.005

**Table 3:** Risk of bias across studies included in this review. Study number 40 (*) was assessed using ROBINS I Tool Cochrane (London, UK).

Study ID	Random Sequence Generation	Allocation Concealment	Blinding of Participants and Personnel	Blinding of Outcome Assessment	Incomplete Outcome Data	Selective Reporting	Other Bias
1 [13]	Low	Unclear	Low	Unclear	Moderate	Low	Moderate
2 [14]	Low	Low	Low	Low	Low	Low	Unclear
3 [15]	Low	Low	Low	Low	Low	Low	Unclear
4 [16]	Low	Low	Low	Low	Low	Low	High
5 [17]	Low	Low	Low	Low	High	Low	High
6 [18]	Low	Low	Low	Low	Low	Low	High
7 [19]	Low	Unclear	Low	Unclear	Low	Low	Moderate
8 [20]	Low	Low	Low	Low	Low	Low	Moderate
9 [21]	Low	Unclear	High	Low	Low	Low	Low
10 [22]	Low	Low	High	Low	Low	Low	Low
11 [10]	Low	Unclear	High	Unclear	Low	Low	Unclear
12 [23]	Unclear	Unclear	High	Unclear	Low	Low	High
13 [24]	Low	Unclear	High	Low	Low	Low	Low
14 [25]	Low	Unclear	High	Low	Low	Low	High
15 [26]	Low	Low	Low	Low	Low	Low	Low
16 [27]	Low	Low	Low	Low	Low	Low	Moderate
17 [28]	Low	Unclear	Low	High	Low	Low	Moderate
18 [29]	Low	Unclear	Low	Unclear	Low	Low	Moderate
19 [30]	Low	Unclear	Low	Low	Low	Low	Moderate
20 [31]	Low	Low	Low	Low	Low	Low	Moderate
21 [32]	Low	Unclear	Low	Low	Low	Low	Moderate
22 [33]	Low	Low	High	Low	Low	Low	Moderate
23 [34]	Low	Low	Low	Low	Low	Low	Moderate
24 [35]	Low	Moderate	High	Moderate	Low	Low	Moderate
25 [36]	Low	Low	High	Moderate	Low	Low	High
26 [37]	Low	Low	Low	Low	Moderate	Low	High
27 [38]	Low	Low	Low	Low	Moderate	Low	High
28 [39]	Low	Low	Low	Low	Moderate	Low	High
29 [40]	Low	Low	Low	Low	Moderate	Low	Moderate
30 [41]	Low	Low	Low	Low	Low	Low	High
31 [42]	Low	Low	Low	Low	Low	Low	Moderate
32 [43]	Low	Low	Low	Low	Low	Low	High
33 [44]	Low	Unclear	High	Low	Low	Low	High
34 [45]	Low	Low	High	Low	High	Low	High
35 [46]	Low	Low	High	Low	Low	Low	High
36 [47]	Low	Low	Low	Low	Low	Low	High
37 [48]	High	High	High	High	Low	Low	Low
38 [49]	Low	Low	Low (Active/Placebo)/High (Diet)	Low	Low	Low	Moderate
39 [50]	Low	Low	Low	Low	Low	Low	Moderate
40 * [51]	Low (due to confounding)	Low (participants selection)	Low (classification of interventions)	Moderate (deviations from intended interventions)	Moderate (missing data)	Low (measurement of outcomes)	Low (selection of the reported result)
41 [52]	Low	Low	Low	Low	Low	Low	Moderate

## Data Availability

No new data created.

## Supplementary Materials

The following supporting information can be downloaded at: https://www.mdpi.com/article/10.3390/life14111485/s1. Tables S1: PRISMA Checklist; Tables S2: Characteristics overview of the 41 included articles.
