# Supplementary material for: Impact of Gut Microbiome Interventions on Glucose and Lipid Metabolism in Metabolic Diseases: A Systematic Review and Meta-Analysis
Source: Life (Basel). 2024 Nov 14;14(11):1485. doi: 10.3390/life14111485 (PMC11595434; doi:10.3390/life14111485)
Supplement: Supplementary file 1 [file life-14-01485-s001.zip › Table S1. PRISMA Checklist.docx]

| Section |  | Included (Yes/No) |
| --- | --- | --- |
| Title | Identify the report as a systematic review and meta-analysis | Yes |
| Abstract | Structured summary including background, objectives, data sources, study eligibility criteria, participants, interventions, and main outcomes | Yes |
| Introduction | Provide rationale for the review; state objectives and hypotheses | Yes |
| Eligibility Criteria | Mention inclusion and exclusion criteria | Yes |
| Search Strategy | Include at least one electronic search, along with a description of all information sources used | Yes |
| Data Collection | \|  \| \| --- \|  \| Describe the method used to extract data from reports, and any processes used to contact authors \| \| --- \| | Yes |
| Risk of Bias Assesement | Specify methods used to assess the risk of bias | Yes |
| Data Synthesis | Specify all statistical methods used, including subgroup analyses and sensitivity analyses | Yes |
| Study Characteristics | Include a table listing characteristics for each included study | Yes |
| Results | Present summary measures, meta-analysis results, and data synthesis | Yes |
| Risk of Bias Across Studies | Discuss the potential impact of risk of bias on the findings | Yes |
| Additional Analysis | Report any other analyses performed (e.g., subgroup or sensitivity analyses) | Yes |
| Disscusion | \|  \| \| --- \|  \| Summarize main findings, limitations, conclusions, and implications for future research \| \| --- \| | Yes |
| Funding | \|  \| \| --- \|  \| Describe the source of funding and potential conflicts of interest \| \| --- \| | Yes |
