# Supplementary material for: Impact of Gut Microbiome Interventions on Glucose and Lipid Metabolism in Metabolic Diseases: A Systematic Review and Meta-Analysis
Source: Life (Basel). 2024 Nov 14;14(11):1485. doi: 10.3390/life14111485 (PMC11595434; doi:10.3390/life14111485)
Supplement: Supplementary file 1 [file life-14-01485-s001.zip › Table S2. Characteristics overview of the 41 included articles.docx]

| Study | Intervention | Study  Design | Sample  Size | Participants | Duration | Methods | Primary Outcomes | Gut microbiota changes |
| --- | --- | --- | --- | --- | --- | --- | --- | --- |
| Depommier et al, 2015 [13] | A. muciniphila supplementation | Randomized, double-blind, placebo-controlled | 32 | Obese, insulin-resistant adults | 3 months | qPCR, 16S rRNA gene sequencing | Insulin sensitivity improved by ~30%, fasting insulin reduced by ~30% | No significant changes in gut microbiome composition were observed |
| Yu et al, 2018 [14] | Fecal Microbiota Transplantation | Randomized, double-blind, placebo-controlled | 24 | Obese adults with mild–moderate insulin resistance | 12 weeks | 16S rRNA, metagenomics | No significant changes in insulin sensitivity, HOMA-IR, HbA1c reduced slightly; greater changes in HDL-c and triglycerides | No significant changes in gut microbiome |
| Allegretti et al, 2018 [15] | Fecal Microbiota Transplantation | Randomized, double-blind, placebo-controlled | 22 | Obese adults | 12 weeks | 16S rRNA, UPLC-MS, short-chain fatty acid analysis | no overall improvement in obesity biomarkers; no change in BMI. | Sustained shift to a lean microbiome profile, with engraftment of OTUs in Faecalibacterium. |
| Kootte et al, 2016 [16] | Fecal Microbiota Transplantation | Randomized, double-blind, placebo-controlled | 38 | Metabolic syndrome patients | 6 weeks | 16S rRNA gene sequencing | Short-term improvement in insulin sensitivity, HbA1c slightly reduced | Altered duodenal and fecal microbiota |
| Mocanu et al, 2021 [17] | FMT + Low-Fermentable Fiber | Randomized, double-blind, placebo-controlled | 68 | Patients with obesity and metabolic syndrome | 12 weeks | 16S rRNA gene sequencing | Significant improvements in insulin sensitivity (HOMA2-IR) and HOMA2-IS at week 6 | Changes in microbial richness and composition, including increases in Bacteroides stercoris and Phascolarctobacterium |
| Rinott et al, 2018 [18] | FMT + Green-Mediterranean diet | Randomized, double-blind, placebo-controlled | 90 | Overweight/obese adults | 6 months | Shotgun metagenomics, 16S rRNA sequencing | 33.6% reduction in weight regain, significant reduction in waist circumference; no significant difference in FPG and HOMA-IR | Significant increase in Akkermansia, Bacteroides vulgatus, and Alistipes putredinis |
| Guevara-Cruz et al, 2018 [19] | Low-fat diet | Randomized, double-blind, placebo-controlled | 146 | Metabolic syndrome patients | 2 months | 16S rRNA gene sequencing | Significant reduction in waist circumference (4-5%), BMI, serum triglycerides (-24%), and leptin (-20-31%) | Increased species richness and diversity , reduced dysbiosis (Prevotella/Bacteroides ratio) |
| Vera et al, 2018 [20] | Functional foods | Randomized, double-blind, placebo-controlled | 81 | T2DM patients | 12 weeks | 16S rRNA gene sequencing | Significant reduction in HOMA-IR, Fasting glucose, HbA1c | Increased Faecalibacterium prausnitzii, Akkermansia muciniphila; Decreased Prevotella copri |
| Meslier et al, 2019 [21] | Mediteranean Diet | Randomized, controlled, parallel-group | 82 | Obese adults | 8 weeks | Metagenomic species pangenome, ultra-high-performance liquid chromatography-mass spectrometry | Significantly reduced total cholesterol, LDL-cholesterol, and HDL-cholesterol | Significant increase in Faecalibacterium prausnitzii, reduction in Bilophila wadsworthia, Roseburia, and Lachnospiraceae taxa; significant decrease in branched-chain fatty acids |
| Kahleova, et al, 2018 [22] | Low-fat vegan diet | Randomized, open parallel-group | 168 | Obese patients | 16 weeks | 16S rRNA gene sequencing using Illumina NextSeq 500 | Insulin sensitivity increased significantly (PREDIM index) | Relative abundance of Faecalibacterium prausnitzii increased |
| Chen et al, 2021 [23] | FMT/Metformin | Randomized, open-label, parallel design | 29 | T2DM patients | 4 weeks | Metagenomic sequencing, qPCR | HOMA-IR significantly reduced, FBG and PBG improved | Microbial richness and Shannon diversity increased after FMT |
| Vrieze et al, 2012 [24] | Allogenic/Autologous Gut Microbiota Infusion | Randomized, controlled, parallel design | 18 | Metabolic syndrome patients | 6 weeks | qPCR, 16S rRNA gene sequencing | Peripheral insulin sensitivity improved by ~45% | Increased gut microbiota diversity in the allogenic group, higher levels of Roseburia intestinalis |
| Wastyk et al, 2018 [25] | Probiotic suppl. | Randomized, double-blind, placebo-controlled | 39 | Metabolic syndrome patients | 10 weeks | 16S rRNA gene sequencing | No significant improvements | Shift in microbiota in responders (Eggerthella, Lachnospira); Akkermansia in non-responders |
| Kanazawa et al, 2019 [26] | Synbiotic suppl. | Randomized, double-blind, placebo-controlled | 88 | Obese patients | 24 weeks | 16S rRNA sequencing, RT-qPCR | higher levels of fasting blood glucose and HbA1c at 12 weeks; no significant changes at 24 weeks; no significant changes in lipid | Significant increases in Bifidobacterium and Lactobacillus, decrease in Akkermansia muciniphila |
| Chambers et al, 2019 [27] | Inulin-propionate ester suppl. | Randomized, double-blind, placebo-controlled | 12 | Obese patients | 42 days | 16S rRNA gene sequencing | Significant improvement in insulin sensitivity | Decrease in diversity, increase in Bacteroides spp with inulin and IPE |
| Tonucci et al, 2014 [28] | Probiotic fermented milk | Randomized, double-blind, placebo-controlled | 50 | T2DM patients | 6 weeks | Fecal SCFA analysis (GC), Cytokine analysis (Luminex xMAP) | Decrease in HbA1c; no significant changes in lipid | Increase in acetic acid (probiotic & control groups), No significant butyric/propionic change |
| Palacios et al, 2016 [29] | Multi-strain probiotic suppl. | Randomised, double-blind, placebo-controlled | 60 | Prediabetes and T2DM patients | 12 weeks | Shotgun metagenomic sequencing | No significant changes in lipid and glucose parameters | Increase in Bifidobacterium breve, Akkermansia muciniphila; no significant changes in species-level beta diversity |
| Wang et al, 2022 [30] | Probiotic suppl. | Randomized, double-blind, placebo-controlled | 59 | T1DM patients | 6 weeks | NGS sequencing (16S rRNA V3-V4 regions) | Significant reduction in HbA1c and fasting glucose | Increased populations of B. animalis, L. salivarius, and Akkermansia muciniphila |
| Wortelboer et al, 2020 [31] | Faecal Filtrate Transplant | Randomized, double-blind, placebo-controlled | 24 | Metabolic syndrome patients | 28 days | Whole Genome Shotgun sequencing (WGS), viral-like particle (VLP) metagenomics | No significant improvement in glucose metabolism (OGTT and AUC); slight improvement in glucose variability | Slight reduction in bacterial and viral richness, non-significant increase in VLP viral richness |
| Moreno-Indias et al, 2015 [32] | Red Wine and De-Alcoholized Red Wine | Randomized crossover trial | 20 | Metabolic syndrome patients | 30 days | PCR-DGGE, qPCR | Significant reduction glucose, triglycerides, and total cholesterol; increase in HDL cholesterol | Increase in Bifidobacterium, Faecalibacterium prausnitzii, Lactobacillus, decrease in Clostridium histolyticum and Escherichia coli |
| Chen et al, 2016 [33] | Yogurt/Milk | Randomized crossover trial | 92 | Metabolic syndrome patients | 24 weeks | 16S rRNA sequencing | Significant reductions in HOMA-IR, fasting insulin, and 2-hour insulin; slight decrease in FBG | Decreased Firmicutes, Clostridiales, Blautia, Ruminococcus, Erysipelotrichaceae; increased Phascolarctobacterium |
| Vulevic et al, 2015 [34] | B-GOS (Prebiotic) | Randomized Crossover Trial | 45 | Metabolic syndrome patients | 12 weeks | 16S rRNA sequencing | Significant reductions in plasma TG, TC and insulin |  |
| Gilijamse et al, 2022 [35] | A. soehngenii suppl. | Randomized Crossover Trial | 24 | Obese patients with insulin resistance | 4 weeks | qPCR, Metagenomics | No changes in insulin sensitivity overall; no significant lipid changes | Significant increase in A. soehngenii proportion and abundanc |
| Balfegó et al, 2013 [36] | Sardine enriched diet | Randomized Crossover Trial | 32 | T2DM patients | 6 months | qPCR | No significant difference in glycemic control; decrease in HOMA-IR observed in both groups, but greater in sardine group | Decreased Firmicutes, decreased Firmicutes/Bacteroidetes ratio in SG; increased Bacteroides-Prevotella group in SG; increased E. coli in both groups |
| Feng et al, 2018 [37] | White common bean extract | Randomized, double-blind, placebo-controlled | 90 | T2DM patients | 4 months | 16S rRNA gene sequencing | HbA1c decreased significantly more in WCBE group vs control; lower fasting and postprandial glucose in WCBE group vs control | Significant enrichment of Bifidobacterium, Faecalibacterium, and Anaerostipes in WCBE group; decrease in Klebsiella, Enterobacteriaceae |
| Li et al, 2021 [38] | Shenlian Formula | Randomized double-blind, placebo-controlled | 31 | T2DM patients | 12 weeks | 16S rRNA gene sequencing | PBG significantly decreased in SL group; FPG, HOMA-IR also improved significantly vs placebo | Significant increase in Bacilli, Lactobacillales (P < 0.05); significant decrease in Clostridiales, Eubacterium hallii group, Subdoligranulum in SL group vs placebo (P < 0.05) |
| Su et al, 2018 [39] | Fecal Microbiome Transplant + Diet | Randomized controlled open-label | 12 | T2DM patients | 90 days | 16S rRNA gene sequencing | FBG decreased significantly in DF group at day 20; HbA1c decreased in both groups | Prevotella increased significantly in both groups, especially in DF; Bifidobacterium, Lactobacillus increased in both groups; Bilophila decreased |
| Hiel et al, 2018 [40] | Inulin Prebiotic | Randomized, single-blind, placebo-controlled | 106 | Obese patients | 3 months | 16S rRNA gene sequencing | HOMA-IR not significantly changed; no changes in lipids | Bifidobacterium increased significantly in prebiotic group (q < 0.001); Desulfovibrio and Butyricimonas decreased; Lactobacillus and Catenibacterium increased |
| Wang et al, 2022 [41] | Probio-X Probiotic | Randomized, double-blind, placebo-controlled trial | 56 | Hyperlipidemic patients | 3 months | Deep metagenomic sequencing | Significant reduction in: TC, LDL-C, HDL-C in probiotic group; No significant changes in TG | Bifidobacterium animalis and Lactiplantibacillus plantarum significantly increased; No major changes in alpha diversity or overall microbiota structure |
| Rabiei et al, 2019 [42] | Synbiotic | Randomized, parallel triple-blind clinical trial | 40 | Metabolic syndrome patients | 12 weeks | - | Significant decrease in FBS, insulin, HOMA-IR, and increased GLP-1 | - |
| Cicero et al, 2021 [43] | Synbiotic | Randomized, double-blind, placebo-controlled trial | 60 | Metabolic syndrome patients | 60 days | - | 23% of synbiotic-treated patients no longer met metabolic syndrome criteria vs. 10% in placebo group | - |
| Storm-Larsen et al, 2022 [44] | Omega-3 PUFA suppl. | Double-blind, placebo-controlled, crossover | 179 | Familial hypercholesterolemia patients | 12 weeks | 16S rRNA gene sequencing | Significant decrease in tryglicerides, total cholesterol and LDL-c | Lower microbial diversity in FH patients compared to controls |
| Eriksen et al, 2016 [45] | Whole Grain Rye vs Wheat + Lignans | Double-blind, placebo-controlled, crossover | 49 | Metabolic syndrome patients | 8 weeks | 16S rRNA gene sequencing | Lower total Cholesterol and LDL | Increased Bifidobacterium, Decreased Lachnospiraceae |
| Bellikci-koyu et al, 2017 [46] | Kefir/Unfermented Milk | Parallel, Randomized Controlled Trial | 22 | Metabolic syndrome patients | 12 weeks | 16S rRNA gene sequencing | Improved insulin sensitivity, reduced HOMA-IR | Increased Actinobacteria, Lactobacillus and Bifidobacterium |
| Haro et al, 2015 [47] | Mediterranean Diet/Low-Fat, High-Complex Carb Diet | Randomized Controlled Trial | 20 | Obese patients | 1 year | 16S rRNA gene sequencing | Increased Insulin Sensitivity; no other significant changes | Increased Roseburia (Med), Increased Prevotella and F. prausnitzii (LFHCC) |
| Nuankham et al, 2022 [48] | L. paracasei suppl. | Randomized, double-blind, placebo-controlled | 22 | Hypercholesterolemic patients | 90 days | 16S rRNA gene sequencing | LDL-C reduction; significant FPG increase (in placebo group) | Increased Flavonifractor, decreased Subdoligranulum, Lachnospiraceae NK4A136, Eubacterium ventriosum, Phascolarctobacterium |
| Ismael et al, 2019 [49] | Mediterranean Diet | Single-arm pilot study | 9 | T2DM patients | 12 weeks | 16S rRNA gene sequencing | HbA1c reduction, HOMA-IR decrease | Increase in Prevotella to Bacteroides ratio, decrease in Firmicutes to Bacteroidetes ratio, increase in bacterial richness and diversity |
| Frias et al, 2022 [50] | Fiber-enriched Nutritional Formula | Randomized, double-blind, placebo-controlled | 192 | T2DM patients | 12 weeks | Metagenomic Sequencing | HbA1c reduction, no FPG reduction | Increased abundance of butyrate-producing species like Roseburia faecis and Anaerostipes hadrus |
| Lee et al, 2022 [51] | B. lactis suppl. | Randomized, double-blind, placebo-controlled | 99 | Obese patients | 12 weeks | - | Significant triglycedies changes; no other significant changes | - |
| Hsieh et al, 2022 [52] | L. reuteri suppl. (ADR1/3) | Randomized, double-blind, placebo-controlled | 74 | T2DM patients | 9 months | Quantitative PCR analysis for gut microbiota, cytokine ELISA, 16S rRNA sequencing | ADR-1 group saw significant reduction in HbA1c; | Increased L. reuteri and Bifidobacterium spp. in ADR-1 and ADR-3 groups, reduction in IL-1β (ADR-3 group) |
| Razmpoosh et al, 2013 [53] | Multi-strain Probiotic suppl. | Randomized, double-blind, placebo-controlled | 60 | T2DM patients | 6 weeks | - | Significant reduction in FPG and significant increase in HDL-C | - |
